# Genome Wide Identification of Orthologous *ZIP* Genes Associated with Zinc and Iron Translocation in *Setaria italica*

**DOI:** 10.3389/fpls.2017.00775

**Published:** 2017-05-15

**Authors:** Ganesh Alagarasan, Mahima Dubey, Kumar S. Aswathy, Girish Chandel

**Affiliations:** ^1^Department of Plant Molecular Biology and Biotechnology, Indira Gandhi Agricultural UniversityRaipur, India; ^2^Department of Agricultural Microbiology, Tamil Nadu Agricultural UniversityCoimbatore, India

**Keywords:** zinc and iron regulated transporters, signal peptide, *Setaria italica*, expression profiling, gene characterization

## Abstract

Genes in the *ZIP* family encode transcripts to store and transport bivalent metal micronutrient, particularly iron (Fe) and or zinc (Zn). These transcripts are important for a variety of functions involved in the developmental and physiological processes in many plant species, including most, if not all, Poaceae plant species and the model species Arabidopsis. Here, we present the report of a genome wide investigation of orthologous *ZIP* genes in *Setaria italica* and the identification of 7 single copy genes. RT-PCR shows 4 of them could be used to increase the bio-availability of zinc and iron content in grains. Of 36 *ZIP* members, 25 genes have traces of signal peptide based sub-cellular localization, as compared to those of plant species studied previously, yet translocation of ions remains unclear. *In silico* analysis of gene structure and protein nature suggests that these two were preeminent in shaping the functional diversity of the *ZIP* gene family in *S. italica*. NAC, bZIP and bHLH are the predominant Fe and Zn responsive transcription factors present in *SiZIP* genes. Together, our results provide new insights into the signal peptide based/independent iron and zinc translocation in the plant system and allowed identification of *ZIP* genes that may be involved in the zinc and iron absorption from the soil, and thus transporting it to the cereal grain underlying high micronutrient accumulation.

## Introduction

Bio-fortification of food crops with Fe and Zn remains a priority area of research. Iron (Fe) and Zinc (Zn) are basic nutrient elements for plants, which assist metabolism and development in plant parts (Haydon and Cobbett, 2007; Samira et al., 2013; Kabir et al., 2014). Plants face challenges in maintaining homeostasis of these two metals, as they may generate highly reactive hydroxyl radicals. The hydroxyl radicals can harm most cell parts, for example, DNA, proteins, lipids and sugars. Zinc serves as an essential basic element in many proteins, including DNA-binding Zn-finger protein (Vallee and Auld, 1990; Rhodes and Klug, 1993), RING finger proteins and LIM domain- containing proteins (Vallee and Falchuk, 1993), whereas iron plays a significant part in electron transfer in photosynthesis and respiration. Thus, plants have developed a firmly controlled framework to balance the uptake and storage of these metal ions (Grotz and Guerinot, 2006; Palmgren et al., 2008; Chandel et al., 2010). Accordingly, Fe and Zn homeostasis in plants have clearly evolved. Since a deficiency of nutrients like Zinc and Iron diminishes the growth of plants, for example influencing rice grain production, both in terms of quantity and quality, whereas over-abundance of Zn and Fe might cause significant toxicity to some biological systems (Pahlsson, 1989; Price and Hendry, 1991). Various metal transporters are available in plants, which pass the metal ions over the layer in the cytoplasm that maintains metal homeostasis (Kambe et al., 2004; Taylor et al., 2004; Colangelo and Guerinot, 2006; Barberon et al., 2014). These include the P-type ATPase (P1B) family, Zinc & Iron-regulated transporter – like Protein (ZIP) (Milner et al., 2012; Thakur et al., 2016), Normal Resistance-Related Macrophage Protein (NRAMP), and the Cation Dissemination Facilitator (CDF) family (Colangelo and Guerinot, 2006; Palmer et al., 2014). It has been reported that OsZIP4, OsZIP5 and OsZIP8 are functional zinc transporters and are localized to the plasma membrane (PM) (Ishimaru et al., 2005; Lee et al., 2010a,b). AtIRT2 is an iron transporter and is localized to the intracellular vesicles, suggesting a crucial role in preventing metal toxicity through compartmentalization and remobilizing iron stores from inner storage vesicles (Vert et al., 2009).

ZRT and the IRT-like protein (ZIP) family has been described far and wide in living beings, including archaea, bacteria, parasites, plants and has been seen with high micronutrient contributor in the endosperm of minor millets. The ZIP family gene proteins comprise 300–500 amino acid residues with six to nine transmembrane domains and besides, a similar membrane topology can transport various divalent cations, including Fe^2+^, Zn^2+^. AtIRT1 was the first individual from the ZIP protein family to be recognized in a yeast mutant defective in iron uptake through functional complementation, and it encodes a major Fe transporter at the root surface in Arabidopsis (Eide et al., 1996; Varotto et al., 2002; Vert et al., 2002).

Minor millets, being nutritiously rich, serve as vital focuses for discovering potential qualities. Foxtail millet is a food security crop in low rain-fed regions. The distinguishing proof of ZIP gene orthologs from micronutrient-rich foxtail millet will unravel their gene reservoir and in the meanwhile will furnish valuable and effective genes for the enhancement of micronutrients in other crops.

A better understanding of the roles and functions of each of the members of the *Setaria italica* ZIP family should lead to new insights into micronutrient homeostasis. Identifying and testing its potentiality in metal transport had been a primary goal of such an effort. Other important features of metal transporters that were focused on in this study are the gene structures of ZIP transporters, whether they have introns or intronless, and the regulation of tissue specific ZIP gene expression. Gaining a better understanding of the *S. italica* ZIP family should also help us better understand micronutrient nutrition in other cereal crop, as the ZIP family of transport proteins is found in all branches of life, including animals, plants, fungi, and protists (Guerinot, 2000). Palmer et al. (2014) reported a genome wide characterization of various ZIP transporters, including spatio-temporal gene expression analysis in one of the closely related C_4_ plant species. To date, no or only a few members of the ZIP family have been characterized in *S. italica* regarding their transport capabilities. We try to put this work into context by stating that such findings will help in reducing malnutrition. Our study will serve as preliminary findings to characterize and functionally validate the single copy orthologs and the functions of signal peptide in plant system.

## Materials and Methods

### Plant Materials and Growth Conditions

The experiment was conducted under protected polyhouse conditions (16 h of photoperiod per day at 30°C) at a geographical location of N 21° 14′ 6.298″E 81°42′ 50.424″. Since the impact of geographical location of plants remain as potential aspect to consider in nutrient accumulation and biological activities, we mentioned the precise location of crop grown area. From the panel of millet genotypes, foxtail millet *Co (Te)7* variety which has greenish purple foliage and yellow grains and little millet cultivar (BL-4, RLM-37 and OLM-203) which has greenish foliage and dark gray grains having high Fe and Zn content was selected. Seeds were treated with 0.1% Bavistin to reduce fungal contamination before sowing. Watering was done once in a week and no nutrient supplementation was given for 3 months of the entire growth period. Completely developed grains were collected from the plants and subjected to micronutrient investigation.

### Elemental Analysis-Atomic Absorption Spectrophotometry

Entire grains of foxtail millet variety and little millet cultivar seeds were physically dehusked using sand paper, followed by the estimation of micronutrients (Stangoulis and Sison, 2008). Fe and Zn concentrations were assessed according to HarvestPlus guidelines^1^ using an atomic absorption spectrophotometer (AAS200) considering tomato leaf powder as standard with minor modifications.

### Database Searches for ZIP Family Genes

All members of the ZIP gene family were exhaustively retrieved from the Gramene database^2^ (Tello-Ruiz et al., 2016) for the two reference plant species *Arabidopsis thaliana*^3^ and *Oryza sativa*.^4^ The retrieved sequences were cross checked with RGAP (Kawahara et al., 2013) and TAIR (Berardini et al., 2015) database for data reliability. The result was confirmed by doing a BLAST analysis against Arabidopsis and Rice genome databases. The accession numbers of published ZIP genes from Arabidopsis and rice along with chromosome coordinates and other information are listed in Supplementary Table S2. ZIP genetic information, including the number of amino acids, cds length and chromosome locations were obtained from the Gramene database. Physical parameters of the ZIP proteins, including isoelectric point (pI), and molecular mass (kDa) were calculated using the compute pI/Mw tool in the ExPASy^5^, with parameters set to ‘average’ (Gasteiger et al., 2005). The gene sequences *viz* CDS, intron, exon and UTR regions were used to mine SSRs in the SSR identification tool^6^.

### Genome Wide Investigation of ZIP Orthologs and Membrane Topology

Here we performed a genome wide survey using OrthoVenn, aimed at identifying orthologs of ZIP genes across three plant species; *O. sativa, A. thaliana* and *S. italica*^7^ (Wang et al., 2015). Thirteen ZIP protein sequences from Rice and 16 from Arabidopsis were used to identify orthologs within a whole genome sequence of foxtail millet. The analysis parameters of OrthoVenn were as follows: cutoff for all-too-all protein similarity comparisons (*E*-value 1*e*-5); and Inflation value (1.5) to generate ortholog clusters using the Markov Cluster Algorithm (Enright et al., 2002). The putative transmembrane topology for each of the ZIP proteins was predicted using PROTTER (version 1.0)^8^.

### Mapping of ZIP Genes on Chromosomes and Gene Structure Prediction

The chromosome positioning of the Arabidopsis, rice and foxtail millet ZIP genes were generated using TAIR^9^, Oryzabase^10^ and Mapchart 2.3 (Voorrips, 2002) respectively. GSDS^11^ was used to predict the exon and intron structures of the individual ZIP genes through alignment of the CDS with their corresponding genomic DNA sequences.

### Molecular Modeling and Phylogenetic Analysis of ZIPs

Multiple sequence alignment of the full length amino-acid sequences of the ZIP proteins were performed by Clustal X2.0.10 (Thompson et al., 1997). An effective phylogenetic tree was developed using the W-IQ-TREE online server (Trifinopoulos et al., 2016) with default options. The SWISSMODEL workspace was used to build homology models of the ZIPs by automated protein structure modeling and the ExPASy web server.

### Motif Analysis of ZIP Protein Sequences and Signal Peptide Prediction

The MEME program software, version 4.9.0 (Bailey and Elkan, 1994) was used to analyze the full length protein sequences of the ZIP genes for motif variation. The motif selection was set to 10 as the maximum number, with a minimum and maximum width of 6 and 50 amino acids, in order to locate the conserved motif. The Distribution of any number of repetitions was considered, while the other factors were of default settings. An upstream sequence of 1KB was subjected to promoter analysis through PlantPAN http://PlantPAN2.itps.ncku.edu.tw (Chow et al., 2015). Protein localization was predicted by TragetP http://www.cbs.dtu.dk/services/TargetP/ (sub-cellular localization) and SignalP http://www.cbs.dtu.dk/services/SignalP/ web servers.

### Tissue Specific *In Silico* Expression Profiling of ZIP Genes in Foxtail Millet

The European Nucleotide Archive^12^ was used to retrieve Illumina RNA-HiSeq reads from four tissues of foxtail millet- namely Root (SRX128223), Stem (SRX128225), Leaf (SRX128224) and Spica (SRX128226), a drought stress library (SRR629694) and its control (SRR629695) (Zhang et al., 2012; Qi et al., 2013). The NGS Toolkit^13^ was employed to filter the reads, and the CLC Genomics Workbench 8^14^ was used to map the reads onto the gene sequences of -*S. italica*. The normalization of the mapped reads was done using the RPKM (reads per kilobase per million) method. Based on the RPKM values, the heat map for tissue-specific expression profile was generated for each gene in all tissue samples using the TIGR MultiExperiment Viewer (MeV v 4.9) software package (Saeed et al., 2003).

### Validation of Functional Orthologs

To validate our *in silico* findings, we have measured the abundance of transcript present in SiZIP orthologous genes. For validation of functional ortholog, foxtail millet seeds were surface sterilized and sown in a pot containing soil and allowed to grow for 15 days at above mentioned growth conditions. Collected tissues were frozen in liquid nitrogen and quickly stored at -80. Total RNA was isolated from the shoots of by using TRIzol reagent, according to the manufacturer’s protocol (Invitrogen, USA). A one step Reverse-transcription reactions involved 1 μl of total RNA by use of the SuperScript III platinum RT-PCR system. The gene-specific primers were designed from the foxtail millet ZIP1, ZIP3, ZIP3, ZIP4, ZIP5, ZIP6, and ZIP7 genes. An RT-PCR program initially started with 55°C for 30 min; 94°C denaturation for 2 min, followed by 40 cycles of 94°C for 15 s, 60–62°C for 30 s and 68°C for 30 s, 68°C annealing for 5 min. Actin gene was used for internal control gene amplification.

### Comparative Expression Analysis of SiZIP Gene Homolog in Other Millet Crop

Two foxtail millet genes were selected based on their expression level. Comparative expression analysis of two foxtail millet ZIP gene homologs (ortholog/paralog) was carried out in other millet crop, i.e., little millet (*Panicum sumatrense*) to find out the existence of SiZIP homologs and its expression level at different tissues. Experimental condition (plant growth condition and expression analysis) in little millet is same as mentioned above for foxtail millet. RNA was isolated from stem, leaf and spica at the panicle emergence stage. All tests were repeated two times, and one of the repeats is shown in the figures. PCR products were resolved by 2.5% agarose gel electrophoresis and stained with EtBr. The gel images were captured using Bio-Rad gel documentation system.

## Results

### Grain Nutrients and ZIP Ortholog Analysis in Foxtail Millet

Fe and Zn estimation revealed that the distribution of zinc and iron contents in foxtail millet varies with the rice. Estimated amounts of 27.19 ± 1.05 μg/g of iron and 40.40 ± 0.23 μg/g of zinc (mean and SE value of the replicated data) were present in *S. italica*.

**FIGURE 1:**
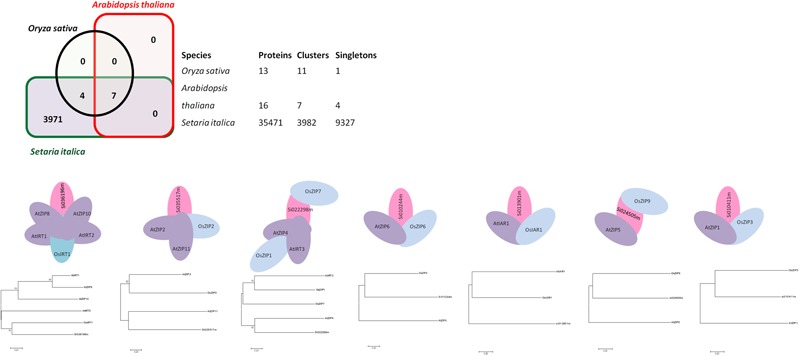
**Ortholgous genes for the ZIP family have been identified among three species viz. Arabidopsis, rice and foxtail millet.** Seven orthologous clusters along with its Phylogenetic tree have been presented.

Genome-wide analysis of orthologous clusters is an important part of comparative genomics study. Identification of overlap among orthologous clusters can enable us to elucidate the role and evolution of proteins across Arabidopsis, rice and foxtail millet species. Orthologs or orthologous genes are clusters of genes in distinct species that originated by vertical descent from a single gene in the last common ancestor. Based on the results of syntenic analysis, precise findings concerning ZIP gene family orthologs were obtained. Well-annotated and well-characterized ZIP family genes from Arabidopsis and rice were used to find orthologs from a whole genome sequence of foxtail millet. Out of 35,471 proteins in foxtail millet, 7 were found to be ZIP ortholog for rice and Arabidopsis (**Figure 1**). Among these three genomes, seven orthologous clusters were obtained. Cluster 1 had a maximum of six proteins, in which Arabidopsis shared four genes (AtIRT1, AtZIP8, AtIRT2 and AtZIP10). Three overlapping orthologous gene clusters were found in the Arabidopsis genome, whereas one was found in the rice genome and none in foxtail millet. Overlapping orthologous genes were distributed on different chromosomes from a single genome in rice and Arabidopsis. Further, there was no multi copy of orthologs found in the foxtail millet genome. The gene IDs for identified orthologs in foxtail millet are given in Supplementary Table S2. Single copy gene clusters are represented in **Figure 1**, and the predicted gene structure of these genes are shown in **Supplementary Figure S1**.

### Chromosomal Distribution of the ZIP Family in Three Species Genomes

**FIGURE 2:**
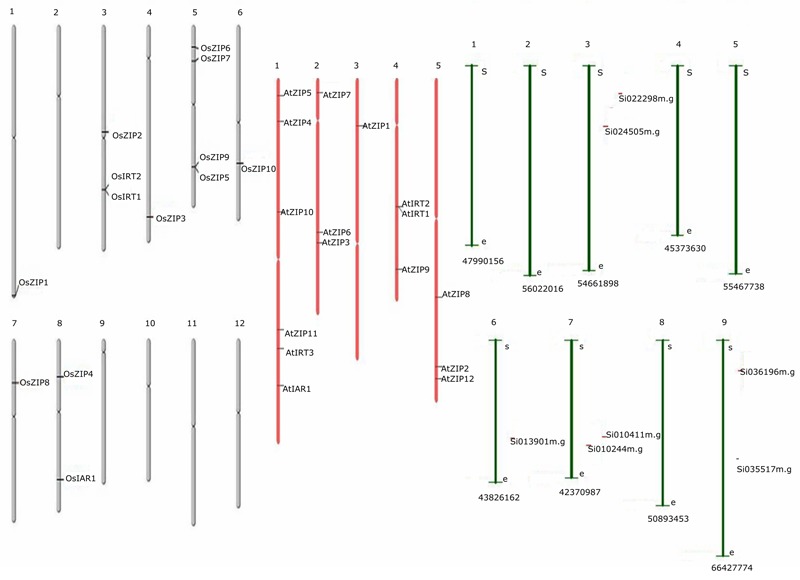
**The distribution of ZIPs across the length of the chromosome, along with its name is mentioned on the right side of every chromosomal segment.** There were a total of 36 genes, 16 from Arabidopsis, 13 from rice and 7 from foxtail millet. Gray color chromosomes indicates rice, whereas pink and green for Arabidopsis and foxtail millet, respectively.

Thirty-six genes were identified as members of the ZIP gene family, including 16 genes in Arabidopsis, 13 in rice and 7 from foxtail millet. Multiple sequence alignment of predicted proteins was shown in **Supplementary Datasheet S1**. Based on these findings, the chromosomal location of ZIP genes was determined for the three species. The results showed an uneven distribution of the 36 ZIP genes on all chromosomes of the three species as shown in **Figure 2**. The genome maps of the ZIP genes showed that AtZIPs were found across all chromosomes of Arabidopsis (Chr. 1,2,3,4, and 5), while OsZIPs were distributed on 7 out of 12 chromosomes (Chr. 1, 3, 4, 5, 6, 7, and 8). In rice, chromosome 5 had the most ZIP genes (4), followed by OsChr3 (3), OsChr8 (2), and OsChr1, 6, and 7 (1-each). AtChr1 (5); AtChr2, 4, and5 (3); and AtChr4 (1) had the ZIP gene distributed discretely in each chromosome. Among the 29 genes, OsIAR1 encoded the longest protein (498 amino acids [aa]), while the shortest (326 aa) was encoded by AtZIP11. The average length of the proteins encoded by the ZIP proteins was 374 aa. The theoretical pi values of the seven proteins (AtZIP3, AtZIP10, OsZIP1, OsZIP3, OZIP4, OsIRT1, OsIRT2) were above 7, showing that they were alkaline, whereas the proteins encoded by the other genes were acidic (<7). The molecular weights of these proteins ranged from 36,021.5 to 53,578.9 Da, with an average of 39,488.42 Da. In the case of foxtail millet seven predicted ZIP genes were located in the chromosome (3, 6, 7, and 9). The detailed parameters are shown in Supplementary Table S2. Although the distributions of these ZIP genes were diverse, their genetic features and biochemical properties tended to be similar.

### Phylogenetic Classification of ZIP Proteins

**FIGURE 3:**
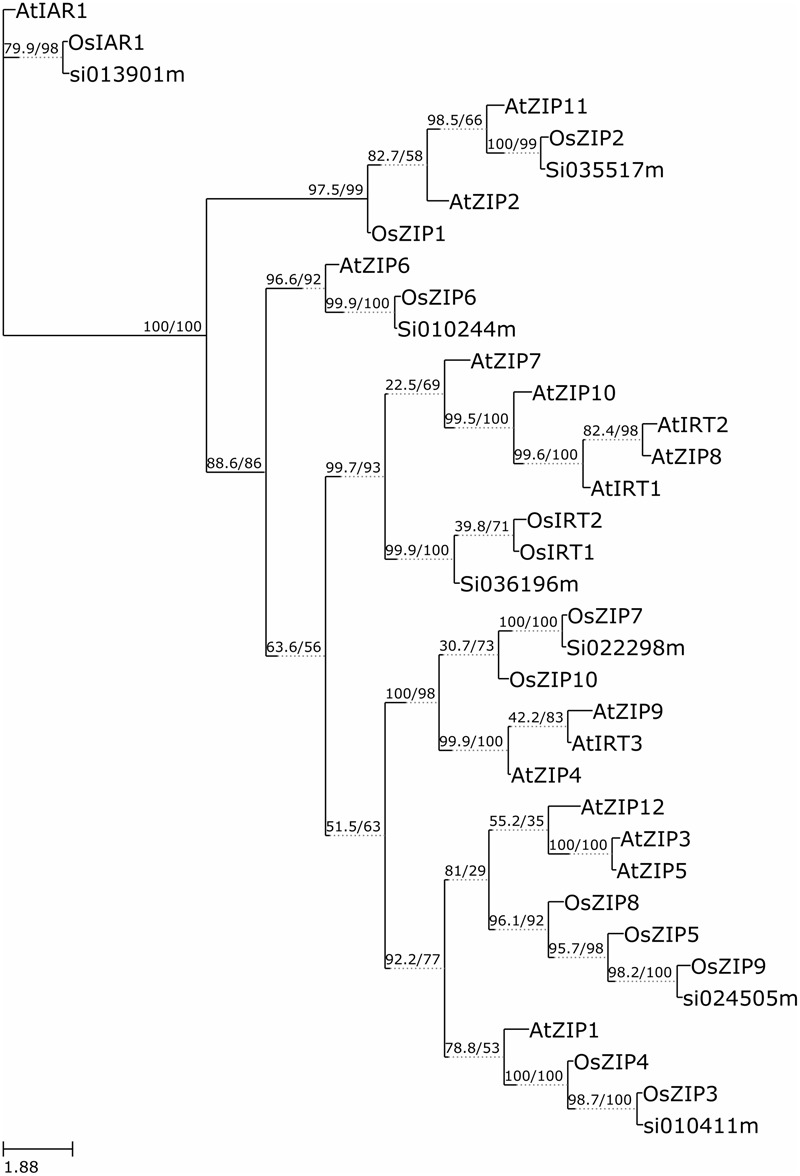
**Molecular Phylogenetic analysis by Maximum Likelihood method.** The evolutionary history was inferred by using the Maximum Likelihood method based on W-IQ-TREE. The bootstrap consensus tree inferred from 1000 replicates is taken to represent the evolutionary history of the taxa analyzed (Felsenstein, 1985). The analysis involved 36 amino acid sequences.

The functional similarity among 36 annotated ZIP genes was explored via phylogenetic analyses of the ZIP protein sequences using the W-IQ-TREE- a maximum likelihood based algorithm. A high bootstrap value suggested a common origin for the ZIP genes of each subgroup. Inspection of the phylogenetic tree topology showed several pairs of ZIP proteins with high homology in the terminal nodes of each subgroup, suggesting that they are putative paralogous pairs (**Figure 3**). The homology modeling structure of SiZIPs is shown in **Supplementary Figure S2**. This outcome upheld the hypothesis that these many sets of paralogous genes may have evolved from a genome duplication event.

### Motif Analysis for Single Copy Gene Orthologs

**FIGURE 4:**
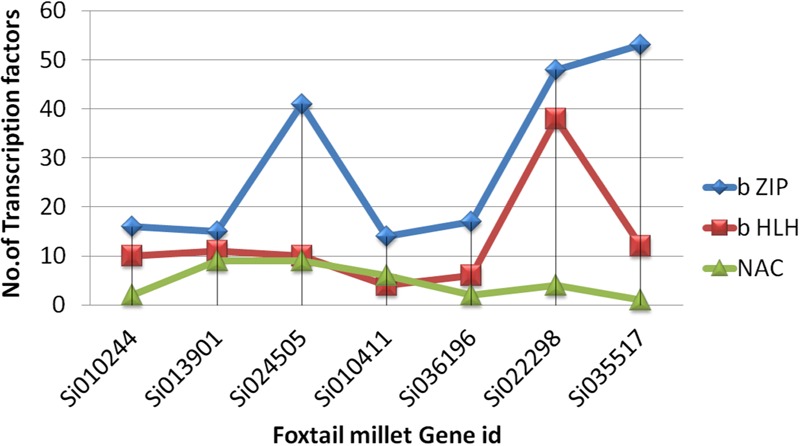
**The distribution of zinc and iron responsive (TF) transcription factors across ZIP in *S. italica***.

Ten conserved motifs were analyzed using MEME software, and the schematic distribution of these 10 motifs among the ZIP proteins is shown in **Supplementary Figure S3**. A common motif distribution was exhibited by the closely related ZIP members as represented by different clusters in the phylogenetic tree; this suggested functional similarities among the ZIP proteins within the same sub-clusters. The distribution of motifs also highlights that the ZIP genes are supposed to be conserved during evolution. The same motif pattern was seen in (OsZIP3, OsZIP9, Si024505g, AtZIP6, OsZIP6 and Si010244). Si013901g had an identical motif compared to OsIAR1 and AtZIP1 but showed positional difference. The AtZIP1 and AtZIP5 motifs distributions were the same, with minor differences, while AtZIP1 had one extra motif. Promoter analyses revealed that the SiZIP family gene has a maximum of b ZIP, b HLH and NAC Fe and Zn responsive transcription factors (**Figure 4**).

### Validation of Functional Orthologs

**FIGURE 5:**
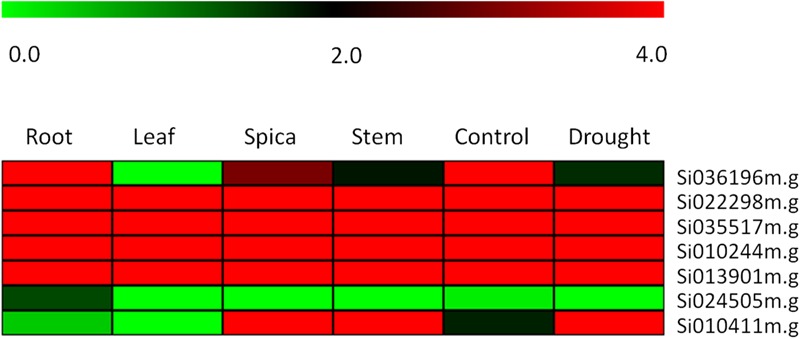
**Heatmap showing the expression pattern of *SiZIP* genes in four tissues, namely root, leaf, spica and stem along with control and drought stress library of *Setaria italica*.** The colored bar at top left represents relative expression value, where 0.0, 2.0, and 4.0 denotes low, medium, and high expression, respectively.

To further confirming our hypothesis and investigate the role of the ZIP family genes in influencing grain Fe and Zn contents, the expression levels of genes were quantified by RNA-seq analysis. Expression of these genes was analyzed among different tissue types and developmental stages to reveal their precise involvement in the transport, remobilization and grain loading of micronutrients. The expression levels of zinc and iron transporter genes were detected by comparing the RPKM value of different genes expressed in the transcriptome of different tissues. In **Figure 5**, RPKM values are represented as a heat map for each identified ZIP transporter gene. The expression of four genes- Si022298m.g, Si035517m.g, Si010244m.g, and Si013901m.g- in four different tissues remains unique and this was found to be maximum with minor variance. In contrast, in Si010411m.g the expression was upregulated in root, leaf, stem and spica tissues. A negligible level of expression was seen in Si024505m.g in all tissues, and exceptionally moderate expression was observed in the root. Meanwhile, the expression of the Si036196m.g gene in leaf tissue was negligible, while a gradual increase was maintained in stem, spica and root tissues’ expression. Interestingly, under stress conditions, the Si036196m.g gene was moderately expressed with maximum expression in control; the opposite was true in Si010411m.g.

**FIGURE 6:**
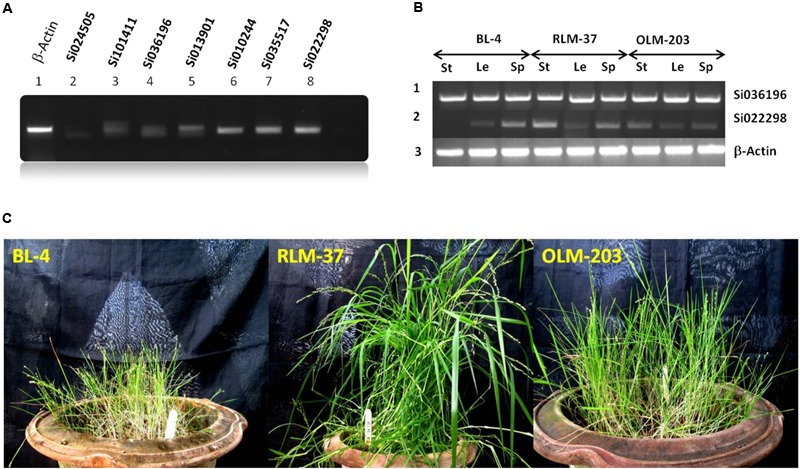
**(A)** RT-PCR expression analysis of seven ZIP genes from *S. italica* in shoot tissues. **(B)** Differential expression analysis of ZIP family gene in little millet. Le, leaf; St, stem and Sp, spica. **(C)** Standing crop view of little millet genotypes.

For wet lab validation, as well as to confirm the SiZIPs’ protein synthesis in SiZIP genes shoot tissues were examined for their transcript abundance. Of seven ZIP genes, four (Si022298m.g, Si035517m.g, Si010244m.g, and Si013901m.g) had relatively good expression levels (**Figure 6**), and they could be used for the bio-fortification process. The list of primers used in this study is given in Supplementary Table S1.

### Comparative Expression Analysis of SiZIP Family Gene Homolog in Other Millet Crop (*Panicum sumatrense*)

Of seven SiZIP genes, we observed four different levels of expression pattern in foxtail millet. Hence, to compare the SiZIP homologs expression data with those of another millet crop, we selected little millet (*P. sumatrense*). Three genotypes BL-4, RLM-37 and OLM-203 (**Figure 6**) were chosen for differential expression analysis using the two SiZIP family gene primers (Si036196 and Si022298). Although Si036196 expression was up regulated in foxtail millet, its expression level was same and maximal in stem, leaf and spica tissues of all three genotypes in little millet. Gene Si02298 exhibited maximum expression in foxtail millet of all tissue types, but in the case of little millet, medium to low levels of expression were observed (**Figure 6**). Homologs of Si036196 and Si022298 had contrasting types of expression level in little millet. Our comparative analysis and results provides a preliminary data for the cloning of potential ZIP genes from non-sequenced crop like little millet.

### Analysis of a Signal Peptide on the Multi-Species (ZIP) Proteins’ N-Terminus

Signal peptides are typically cleaved from the mature proteins during transport and/or processing through the endoplasmic reticulum. When comparatively analyzed with cleavable signal peptides in other organisms, the N-termini of ZIP proteins from rice, Arabidopsis, and foxtail millet showed the presence of signal peptide regions in plant iron/zinc transport proteins. The predicted sub- cellular localization and signal peptide cleavage site of ZIP proteins are presented in Supplementary Table S2 and the signal peptide is shown in **Supplementary Figure S4**. From the plant species studied here, it was posited that most transport proteins have signal peptides, and this suggests a role in the active transport of ion molecules, as supported by the creation of mutant lines of signal peptide in *Malus xiaojinensis* (Zhang et al., 2014).

## Discussion

The ZIP family genes have been identified in many plants like Arabidopsis and rice. These genes are responsible for the transport of Zn and Fe and are known to play a role in Mn transport. The complete genome sequence of foxtail millet has allowed researchers to identify and characterize various gene families in foxtail millet (Lata et al., 2014; Mishra et al., 2014; Yadav et al., 2014; Muthamilarasan et al., 2015a,b). Although ZIPs have been characterized in many plants, to the best of our insight, there were few or no reports on the functional characterization of the ZIPs in foxtail millet, though it has high micronutrients. The nutritional profile (Muthamilarasan et al., 2016) and genetic improvement of cereal crops using foxtail millet genome has been extensively reviewed (Muthamilarasan and Prasad, 2015).

In our study, we identified and characterized seven SiZIP from *S. italica*. Comparative analysis of SiZIPs with other species showed that foxtail millet has fewer orthologous genes (7) then rice (8) or Arabidopsis (12). The protein properties of SiZIP, AtZIP and OsZIP revealed many differences in amino acid length, isoelectric point, molecular weight and trans-membrane domains. In addition, the proteins had different signaling peptides, which could result from the presence of novel splice varients.

To analyze the evolutionary relationship of SiZIPs, a fair phylogenetic tree comprising ZIPs from Arabidopsis and rice was constructed. It was found that predicted amino acids were closely related to other plant species and existed as an ortholog in Arabidopsis and rice.

Meanwhile, we investigated the expression of genes in *S. italica* in response to stress and normal growth conditions. We hypothesized that genes that show differential expression under various stress exposures are more likely to be involved in metal homeostasis. Most of these genes are differentially expressed due to downstream changes in the physiological status of plants as a result of changes in metal homeostasis, although a couple of genes are directly involved in regulating metal homeostasis. The expression patterns of SiZIP genes reflect their diverse functions during Zn and or Fe translocation. It has been reported that the ZIP genes display various expression profiles due to tissue specificity and in response to fluctuating environmental Zn and Fe conditions. For instance, OsZIP7a was induced in Fe-deficient root, while OsZIP8 was stimulated in Zn-deficient shoots and roots (Yang et al., 2007). Histochemical confinement analysis showed that the mRNA of OsZIP4 was more in the vascular bundles of leaves and roots and phloem cells of the stem and the meristems (Ishimaru et al., 2005). Hence, these results demonstrated that SiZIP genes encode Zn or Fe transporters and have various functions associated with uptake and translocation, detoxification and storage of Zn and or Fe in plant cells.

Homology modeling of SiZIP revealed that proteins with similar patterns are not equally expressed (Si036196m.g, Si024505m.g and Si010411m.g), whereas proteins with different patterns were found to be equally expressed, irrespective of tissue type (Si022298m.g, Si035517m.g, Si010244m.g and Si013901m.g), which could be due to effects of gene homologs present in the same species. Comparative expression analysis of SiZIP with little millet showed that Si036196 gene homolog can be further cloned for functional characterization. Sequencing nutritionally rich crops like little millet will provide a better platform to enhance the micronutrient content in other cereal crops.

SSR identification in SiZIPs revealed that SiZIP2-Intronic (TC)_6_, SiZIP3-CDS (CCA)_5_, SiZIP5-Intronic (TC)_5_ and SiZIP7-Cds (GC)_5_ have repeat motifs in their CDS and intronic regions Supplementary Table S3. These SSR repeats could be used for allele mining of the respective genes in Marker Assisted Selection (MAS) crop breeding programs.

Trafficking of the multi-pass PM proteins such as ZIP typically requires an N-terminal signal peptide (Blobel and Dobberstein, 1975; Martoglio and Dobberstein, 1998). A multi pass integral PM protein, *S. italica* ZIP family is predicted to comprise 5 to 9 TMs, with a putative N-terminal signal peptide of 1–30 amino acids with cleavage site ranging from 25 to 30 amino acids. Here we hypothesize that a maximum of ZIP genes in plants might transport and maintain homeostasis with the help of signal peptide present in their N-terminal end. Altogether, our study provides a computational framework for *in silico* characterization of any particular gene family, which can be utilized in MAS breeding and genetic engineering of field crops. Care must be taken before selecting a gene of interest in downstream analysis. Further the effect of homologs in the transgenic plants can be minimized by transfer of single copy orthologs.

## Conclusion

Our results propose that SiZIP genes encode functional Zn and or Fe transporters that may regulate the uptake, translocation and storage of divalent metal ion in plant cells and mainly endosperm. Transcriptome analysis hints the sustained expression level of identified gene orthologs among spica, leaf, stem and root tissues. The present study provides new insights into the evolutionary relationship and putative functional divergence of the ZIP gene family during the growth and development of rice, Arabidopsis and foxtail millet. *In silico* functional characterization viz., expression analysis, transcription factor mining, homology modeling, phylogenetic analysis and wet lab validation of SiZIP family showed that Si02298m.g, Si03557m.g, Si010244m.g and Si013901m.g genes could serve as potential genes in Fe and Zn biofortification. Comparative expression analysis of SiZIP homolog in little millet showed the existence of potential ZIP genes for biofortification. Further characterization of identified orthologs in *S. italica* and their functional validation in a panel of genotypes under varying nutrient supplement will help in divulging new sources of nutritionally important genes for improvement of staple food crops.

## Author Contributions

GC and GA conceived and designed the experiment. GA wrote the manuscript, performed the *in silico* experiments and MD maintained plant materials and has done the nutritional analysis and wet lab validation. All the authors have revised the final draft of the manuscript.

## Conflict of Interest Statement

The authors declare that the research was conducted in the absence of any commercial or financial relationships that could be construed as a potential conflict of interest. The reviewer VB and handling Editor declared their shared affiliation, and the handling Editor states that the process nevertheless met the standards of a fair and objective review.

## Footnotes

1 http://www.harvestplus.org/content/crop-sampling-protocols-micronutrient-analysis

2 http://www.gramene.org

3 https://www.arabidopsis.org/Blast/index.jsp

4 http://rice.plantbiology.msu.edu/analyses_search_blast.shtml

5 http://www.expasy.org/tools/

6 http://www.gramene.org/db/markers/ssrtool

7 http://probes.pw.usda.gov/OrthoVenn

8 http://wlab.ethz.ch/protter/start/

9 https://www.arabidopsis.org

10 http://viewer.shigen.info/oryzavw/maptool/MapTool.do

11 http://gsds.cbi.pku.edu.cn/

12 http://www.ebi.ac.uk/ena

13 http://www.nipgr.res.in/ngsqctoolkit.html

14 https://www.qiagenbioinformatics.com/

## Supplementary Material

FIGURE S1 **Intron-exon structure of SiZIPs as predicted by GSDS**.

FIGURE S2 **Homology modeling of 7 SiZIPs as predicted by SWISS-MODEL given in 3-D structure**.

FIGURE S3 **Motif analysis has been done among the 12 single copy ortholog ZIP gene family members of Arabidopsis, rice and foxtail millet.** The different color bars in one gene indicate different motifs.

FIGURE S4 **Membrane topology of ZIP proteins across rice, Arabidopsis and foxtail millet.** Red color amino acids represent signal peptides of respective protein.

DATASHEET S1 **Multiple sequence alignment of predicted amino acid sequences of SiZIP, AtZIP and OsZIP proteins**.
