# Supplementary figures and images for: Genome Wide Identification of Orthologous ZIP Genes Associated with Zinc and Iron Translocation in Setaria italica
Source: Front Plant Sci. 2017 May 15;8:775. doi: 10.3389/fpls.2017.00775 (PMC5430159; doi:10.3389/fpls.2017.00775)

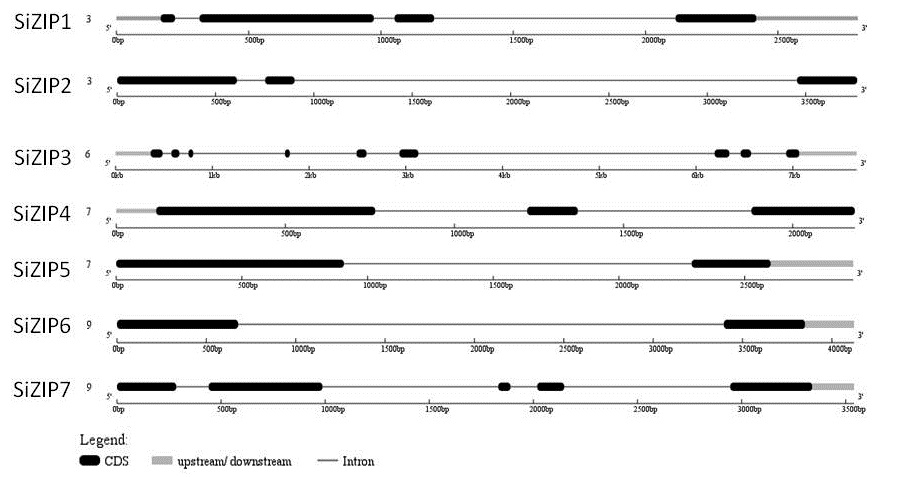

Supplement: FIGURE S1 — Intron-exon structure of SiZIPs as predicted by GSDS. [file Image_1.jpg]

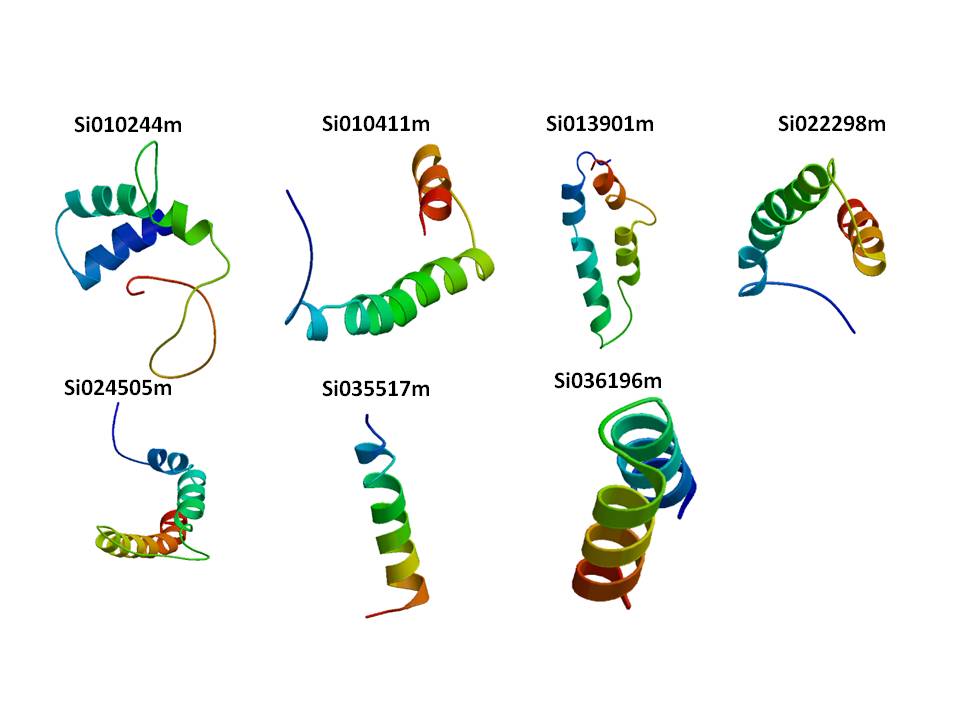

Supplement: FIGURE S2 — Homology modeling of 7 SiZIPs as predicted by SWISS-MODEL given in 3-D structure. [file Image_2.jpg]

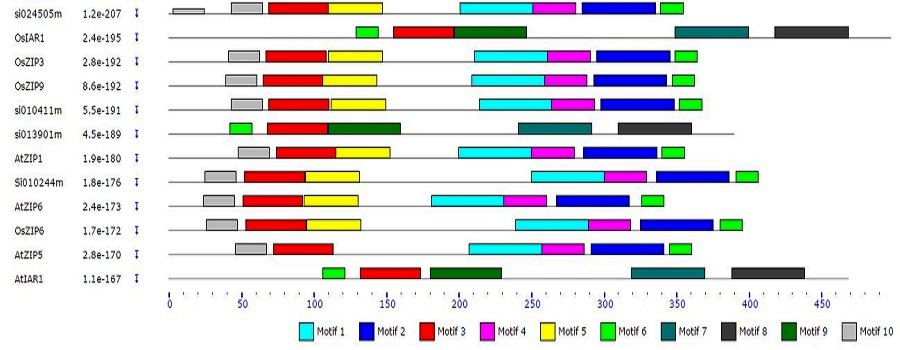

Supplement: FIGURE S3 — Motif analysis has been done among the 12 single copy ortholog ZIP gene family members of Arabidopsis, rice and foxtail millet. The different color bars in one gene indicate different motifs. [file Image_3.jpg]

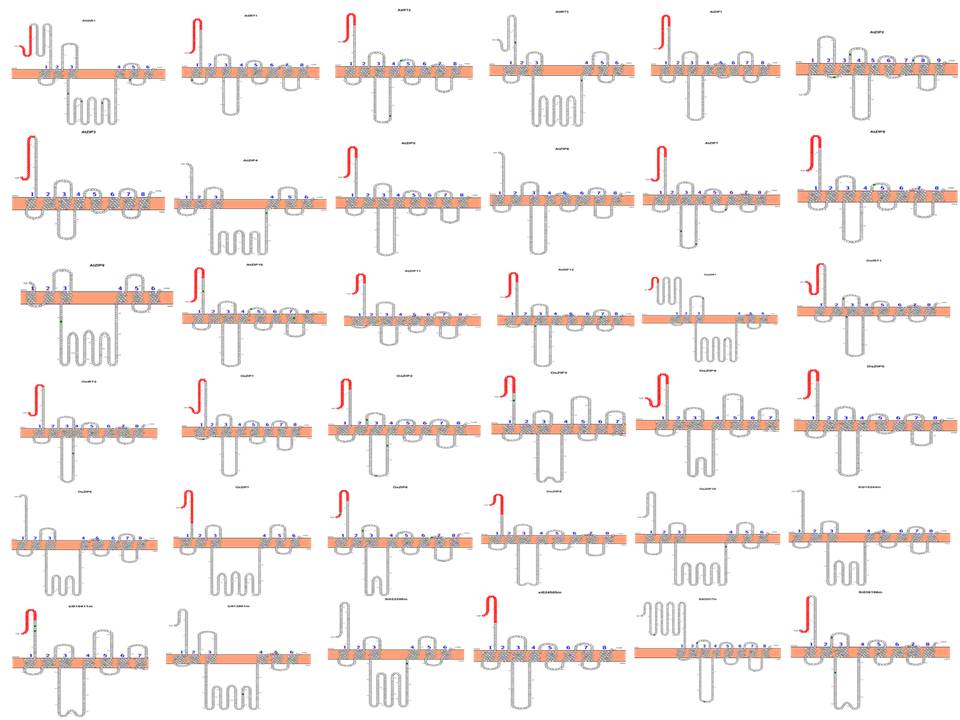

Supplement: FIGURE S4 — Membrane topology of ZIP proteins across rice, Arabidopsis and foxtail millet. Red color amino acids represent signal peptides of respective protein. [file Image_4.jpg]
