# Supplementary material for: Genome Wide Identification of Orthologous ZIP Genes Associated with Zinc and Iron Translocation in Setaria italica
Source: Front Plant Sci. 2017 May 15;8:775. doi: 10.3389/fpls.2017.00775 (PMC5430159; doi:10.3389/fpls.2017.00775)
Supplement: DATASHEET S1 — Multiple sequence alignment of predicted amino acid sequences of SiZIP, AtZIP and OsZIP proteins. [file Data_Sheet_1.pdf]

This website uses cookies. By continuing to browse this site, you are agreeing to the use of our site cookies. To find out more, see our Terms of Use.

Clustal Omega

Results for job clustalo-I20160602-130441-0360-21703108-pg

CLUSTAL O(1.2.1) multiple sequence alignment

```
AtIAR1          ------------------------------------------------------------
OsIAR1          ------------------------------------------------------------
si013901m       ------------------------------------------------------------
AtZIP11         ------------------------------------------------------------
OsZIP2          ------------------------------------------------------------
Si035517m       MTATPQRIIHGGHNWERPERPGRRLLTDFPRPNPTPSLSVLPVYRPPGTPFPWLPAVPFS
AtZIP2          ------------------------------------------------------------
OsZIP1          ------------------------------------------------------------
AtZIP6          ------------------------------------------------------------
OsZIP6          ------------------------------------------------------------
Si010244m       ------------------------------------------------------------
AtZIP7          ------------------------------------------------------------
OsIRT2          ------------------------------------------------------------
OsIRT1          ------------------------------------------------------------
Si036196m       ------------------------------------------------------------
AtZIP10         ------------------------------------------------------------
AtIRT2          ------------------------------------------------------------
AtIRT1          ------------------------------------------------------------
AtZIP8          ------------------------------------------------------------
OsZIP7          ------------------------------------------------------------
Si022298m       ------------------------------------------------------------
AtZIP9          ------------------------------------------------------------
OsZIP10         ------------------------------------------------------------
AtIRT3          ------------------------------------------------------------
AtZIP4          ------------------------------------------------------------
AtZIP12         ------------------------------------------------------------
AtZIP3          ------------------------------------------------------------
AtZIP5          ------------------------------------------------------------
AtZIP1          ------------------------------------------------------------
OsZIP4          ------------------------------------------------------------
OsZIP3          ------------------------------------------------------------
si010411m       ------------------------------------------------------------
OsZIP8          ------------------------------------------------------------
OsZIP5          ------------------------------------------------------------
OsZIP9          ------------------------------------------------------------
si024505m       ------------------------------------------------------------


AtIAR1          --------------------------------------------------------MSFS
OsIAR1          ------------------------------------------------------------
si013901m       ------------------------------------------------------------
AtZIP11         ------------------------------------------------------------
OsZIP2          ---------------------------------------------------------MAG
Si035517m       HLPRVIARTTIAARPPARISHPPTALRAPRNLAHLPRPLSLTIRESCLVHQPPPLHARHG
AtZIP2          --------------------------------------MA--L-----------------
OsZIP1          ----------------------------------MARTMT--M-----------------
AtZIP6          ------------------------------------------------------------
OsZIP6          ------------------------------------------------------------
Si010244m       ------------------------------------------------------------
AtZIP7          ---------------------------------------------------------MAY
OsIRT2          ------------------------------------------------------M-MMSS
OsIRT1          ---------------------------------------------------------MAT
Si036196m       ------------------------------------------------------------
AtZIP10         --------------------------------------------------------MTKS
AtIRT2          ------------------------------------------------------------
AtIRT1          --------------------------------------------------------MASN
AtZIP8          ---------------------------------------------------------MAT
OsZIP7          ------------------------------------------------------------
Si022298m       ------------------------------------------------------M-ALAG
AtZIP9          ------------------------------------------------------------
OsZIP10         ------------------------------------------------------------
AtIRT3          --------------------------------------MF--FV-DVLWK-LVPL-YLFG
AtZIP4          ------------------------------------------------------------
AtZIP12         ---------------------------------------------------------MSR
AtZIP3          ------------------------------------------------------------
AtZIP5          ------------------------------------------------------------
AtZIP1          ---------------------------------------------------------MSE
OsZIP4          ---------------------------------------------------------MDA
```

```
OsZIP3         ---------------------------------------------------------MGA
si010411m      ---------------------------------------------------------MAA
OsZIP8         ------------------------------------------------------------
OsZIP5         ------------------------------------------------------------
OsZIP9         ------------------------------------------------------------
si024505m      ------------------------------------------------------------


AtIAR1         LRK-------LLVPIL-VLVLFLDLCVESG----------FSQSTPARDDHVHHHGG---
OsIAR1         MRG----------GLL-VL-LLLTAAVAGGGGHEGSSSCPFHAHDKPHDDHHHGHGHGHG
si013901m      ------------------------------------------------------------
AtZIP11        -----MS---RSLVFFFLF--LVLVVPC--LSHGTGG-----------------------
OsZIP2         GRGARAS---LHLHLAWL---CAFATTA--WAHGGGGG---------------------G
Si035517m      SRHHQPP---PHLPLR-R---RSAATTA--WAHGGGG----------------------G
AtZIP2         -SS--KT---LKSTLFFLSIIFLCFSLI--LAHGGIDDG--------------------D
OsZIP1         -RV--SS---LLV--AVVLLAALSFQAC--SGHGGINDG--------------------D
AtZIP6         -----------------------------------MASC--------------------V
OsZIP6         ---------------------------------MSGTGC--------------------F
Si010244m      ---------------------------------MSGTGC--------------------F
AtZIP7         SK-ACY----KLTTITILLLSFTLPSLA----GNAENAD--------------------V
OsIRT2         SQ-T------PVRIAFVFLVILAATDAH-----SDHRTP--------------------P
OsIRT1         PR-TLVPILPPVAALLLLLVAASSIPILAAAQPA--DAC--------------------G
Si036196m      --------MSSRTHLLAVLVLLAAAPFVADAQPPAADPA--------------------A
AtZIP10        HV-IF---S-ASIALFLLLSIS--------HFPGALSQS--------------------N
AtIRT2         -M-AT---T-KLVYILLILFTF--------TVSPAISTA--------------------P
AtIRT1         SA-LL---M-KTIFLVLIFVSF--------AISPATSTA--------------------P
AtZIP8         TT-QH---M-NQIFLVLLLISF--------AISPAISTV--------------------P
OsZIP7         -M----E------R----FVQFLRRGNGLMAASLAAGSC--------------------A
Si022298m      LR----R------H----AGQFLSTSNELMAASLSTATC--------------------A
AtZIP9         ------------------------------------------------------------
OsZIP10        MESSS-------------SSSYIPFI-RQIAASVSAASC--------------------D
AtIRT3         SETKSLS------A----TESILQIVPEAMAATSSNVLC---------------------
AtZIP4         -----------------------------MASSTTKILC---------------------
AtZIP12        FR-KT-----LVSAFVLCLVIFPL------LVSAAEEEN--------------------Q
AtZIP3         -M-KTKNVKL-LFFFFSVSLLLIAVVNAAEGHSHGGPKC--------------------E
AtZIP5         MR-ITQNVKLLLFFFFFISFLFIAV-------SAGESKC--------------------E
AtZIP1         CG--CFSATTMLRI---CVVLIICL-------HMCCASS--------------------D
OsZIP4         MR-QSTPR-AMLLL---CAVLMLAVAPPGAATAAAVAGC--------------------E
OsZIP3         KK-HT-----LQVL---PWLLLF-------AQHTAASAC--------------------D
si010411m      VK-HT-----FKVL---SWLLLF-------AQLAFASTS--------------------N
OsZIP8         MR-TNTTATVLLAA---AVALLL-----ATAARGDGGDG--------------------G
OsZIP5         MA--TAA----MTK---VFVLLFLVAACYLPAHAAAAEC--------------------D
OsZIP9         MA-FDLK----LTA-----CL-LL----AVFSLAAAADC--------------------E
si024505m      MA-PDLK----LSA---VFCLLAV----ASLPLLAVADC--------------------E


AtIAR1         -GCSHSHDHD---HDHDHDHHVKKTTAKVEMKLPEELAEEEDMRLCGFGPCLHDHDH---
OsIAR1         HSCGGGGDDSHHVHHHHHGHGHGHDGGIQRRLLPEELAEEADLELDSFGDHHHHHHHHHE
si013901m      ------------------------------------MAEEADLELESFGFDEHDHAHHHH
AtZIP11        D----HD-DDE-------ASHVKS------------------------------------
OsZIP2         D----SDADAD-------GGGEGK------------------------------------
Si035517m      D----PDPDAG-------AGDSPK------------------------------------
AtZIP2         EEEETNQPPPA-------TGTTTV------------------------------------
OsZIP1         GQVDAPATPA------------SS------------------------------------
AtZIP6         TGT-E---AAI-------RAAACR------------------------------------
OsZIP6         PAG-E---MAA-------VARVCR------------------------------------
Si010244m      PDG-G---PA--------GSRACR------------------------------------
AtZIP7         SECK----AES-------GDLSCH------------------------------------
OsIRT2         PACG----GAA-------VGGECH------------------------------------
OsIRT1         GAPD----QAA-------ADGACH------------------------------------
Si036196m      DACA----DPS-------VDGACH------------------------------------
AtZIP10        KDCQ-----SK-------SNYSCI------------------------------------
AtIRT2         EHCD-----SG-------FDNPCI------------------------------------
AtIRT1         EECG-----SE-------SANPCV------------------------------------
AtZIP8         KECE-----TD-------STDSCI------------------------------------
OsZIP7         EEV-----AKA-------EGAGCR------------------------------------
Si022298m      EEM-----QKA-------EGGGCR------------------------------------
AtZIP9         ------------------------------------------------------------
OsZIP10        AVVGG---GGD-------KDEECR------------------------------------
AtIRT3         --------NAS-------ESDLCR------------------------------------
AtZIP4         --------DAG-------ESDLCR------------------------------------
AtZIP12        --CGG---SKG---------GSAA------------------------------------
```

```
AtZIP3         --CSH---EDD-----------HE------------------------------------
AtZIP5         --CSH---EDD-----------EA------------------------------------
AtZIP1         --CTS---HDD--------PVSQD------------------------------------
OsZIP4         --CGN---AAA-------AAVAGE------------------------------------
OsZIP3         --CAN---TTD---------GA--------------------------------------
si010411m      --CTN---ATD---------GTET------------------------------------
OsZIP8         --CGK---EDA---------AAGR------------------------------------
OsZIP5         --CA----TD----------TAGR------------------------------------
OsZIP9         --CQ----PS----------DEGH------------------------------------
si024505m      --CE----AST---------DEDS------------------------------------


AtIAR1         -----------ESSSTLTGFALWLNALGCSLLVSLASLICLVLLPIMFVQG-------KP
OsIAR1         HHHHDHHHGHGDFQAELSPLGMWLSAMGCSLLVSMASLVCLVLLPVIFFQG-------KP
si013901m      HHHGHHH---HHDGMETSPMGVWLSAMGCSLLVSMASLICLVLLPVIFFQG-------KP
AtZIP11        -----------SDLKSKSLISVKIACLVIIFVLT-FISGVSPYFL-------------KW
OsZIP2         -----------PDLRARGLVAAKLWCLAVVFAGT-LAGGVSPYFM-------------RW
Si035517m      -----------PDLRARGLVAAKLWCLAAVFAGT-LLGGVSPYFM-------------RW
AtZIP2         -----------VNLRSKSLVLVKIYCIIILFFST-FLAGVSPYFY-------------RW
OsZIP1         -----------SGVRSKGLIAVKVWCLVILLVFT-FAGGVSPYFY-------------RW
AtZIP6         -----------DGEEASH---LKIVAVFAIFLTS-VFGVWGPVLLAKYFHG------KPL
OsZIP6         -----------DGAAAAR---LKTGSLLAILVAS-AVGICLPVALTGAFRG------KAG
Si010244m      -----------DGAAAAR---LKTGSLLAILVAS-AVGICLPVALTRAFRG------GPN
AtZIP7         -----------NNKEAQK---LKIIAIPSILVAS-MIGVSLPLFSRSIPAL-------GP
OsIRT2         -----------SVARALR---LKLIAIPAILAAS-VAGVCLPLFARSVPAL-------RP
OsIRT1         -----------DVPRALR---LKLIAIPTILVSS-VVGVCLPLLSRSVPAL-------RP
Si036196m      -----------NVPKALR---LKLIAIPTILVAS-VIGVCLPLFSRSIPAL-------RP
AtZIP10        -----------DKNKALD---LKLLSIFSILITS-LIGVCLPFFARSIPAF-------QP
AtIRT2         -----------NKAKALP---LKIVAIVAILTTS-LIGVTSPLFSRYISFL-------RP
AtIRT1         -----------NKAKALP---LKVIAIFVILIAS-MIGVGAPLFSRNVSFL-------QP
AtZIP8         -----------DKTKALP---LKIVAIVAILVTS-MIGVAAPLFSRYVTFL-------HP
OsZIP7         -----------DDAAALR---LKGVAMATILVAG-VVGVGLPLAGRKRRAL-------RT
Si022298m      -----------DDAVALR---LKEVAMAAILVAG-VLGVGLPLAGRKRRAL-------RT
AtZIP9         --------------------------MASILISG-AAGVSIPLVGTLLPL----------
OsZIP10        -----------DEAAALR---LKMVAVAAILIAG-AAGVAIPLVGRRRRGGGGGGGGGAS
AtIRT3         -----------DDSAAFL---LKFVAIASILLAG-AAGVTIPLIGRNRRFL-------QT
AtZIP4         -----------DDSAAFL---LKFVAIASILLAG-AAGVAIPLIGRNRRFL-------QT
AtZIP12        -----------EKASALK---YKIIAFFSILIAG-VFGVCLPIFG-----L-------KT
AtZIP3         -----------NKAGARK---YKIAAIPTVLIAG-IIGVLFPLLGKVFPSL-------RP
AtZIP5         -----------NKAGAKK---YKIAAIPSVLAAG-VIGVMFPLLGKFFPSL-------KP
AtZIP1         -----------EAEKATK---LKLGSIALLLVAG-GVGVSLPLIGKRIPAL-------QP
OsZIP4         -----------DARGALR---LKLVAIASILAAG-AAGVLVPVLGRSFAAL-------RP
OsZIP3         -----------DRQGAMK---LKLIAIASILAAG-AAGVLVPVIGRSMAAL-------RP
si010411m      -----------DKLGAMK---LKLIAIASILTAG-AAGVLVPVLGRSMAAL-------NP
OsZIP8         -----------DRARARG---LKIAAFFSILVCG-ALGCGLPSLGRHVPAL-------RP
OsZIP5         -----------DKAQALR---LKVIAIFCILAGS-TVGAALPSLGGRFPAI-------QP
OsZIP9         -----------DAAKSRT---LKVIAIFCILVGS-SAGCAIPSLGRRFPAL-------RP
si024505m      -----------DKARALT---LKIVAIFCILVAS-SVGCAIPSLGRRFPAL-------RP
                                         .   :

AtIAR1         SKWFVDSLALFGAGAMLGDAFLHQLPHAFGGGHSHSNDHHENHDHHDHSHSDSPSHSHSI
OsIAR1         SKTMVDCLAIFGAGAMLGDSFLHQLPHAFGGGHSHTHDHQ------NHNHSHEHSHAHSL
si013901m      SKAMVDALAVFGAGAMLGDSFLHQLPHAFGGGHSHSHDHE------GHDHAQEHAHAHSL
AtZIP11        SQGFLVLGTQFAGGVFLATALMHFLSDADETFRGLLTAEGES----------EPSPAYP-
OsZIP2         NDAFLALGTQFAGGVFLGTAMMHFLADANETFADLL-----------------PGTAYP-
Si035517m      NEAFLALGTQFAGGVFLGTALMHFLSDANETFGDLL-----------------PGSAYP-
AtZIP2         NESFLLLGTQFSGGIFLATALIHFLSDANETFRGLK------------------HKEYP-
OsZIP1         NESFLLLGTQFAAGVFLGTALMHFLADSTSTFKGLT------------------TNQYP-
AtZIP6         YDKAILVIKCFAAGVILSTSLVHVLPEAFESLAD-C-QVSSR----------HPWKDFP-
OsZIP6         YARGLLLVKCYAAGVILSTSLVHVLPDAHAALAD-C-AVATR----------RPWRDFP-
Si010244m      YARGLLLVKCYAAGVILSTSLVHVLPDAQAALAD-C-AVATR----------RPWRDFP-
AtZIP7         DREMSVIVKTLASGVILATGFMHVLPDSFDDLTSKC-LPED------------PWQKFP-
OsIRT2         DGGLFAVVKAFASGVILGTGYMHVLPDSFNDLTSPC-LPRK------------PWSEFP-
OsIRT1         DGGLFAVVKAFASGVILATGYMHVLPDAFNNLTSPC-LPRK------------PWSEFP-
Si036196m      DRNLFVIVKAFASGVILATGYMHVLPDSFNNLSSPC-LPRK------------PWAEFP-
AtZIP10        EKSHFLIVKSFASGIILSTGFMHVLPDSFEMLSSPC-LNDN------------PWHKFP-
AtIRT2         DGNGFMIVKCFSSGIILGTGFMHVLPDSFEMLSSKC-LSDN------------PWHKFP-
AtIRT1         DGNIFTIIKCFASGIILGTGFMHVLPDSFEMLSSIC-LEEN------------PWHKFP-
AtZIP8         DGKIFMIIKCFASGIILGTGFMHVLPDSFEMLSSPC-LEDN------------PWHKFP-
OsZIP7         DSAAFVAAKAFAAGVILATGFVHMLHDAEHALSSPC-LPAH------------PWRSFP-
Si022298m      DSSAFRAAKAFAAGVILATGFVHMLHDAQHALSSPC-LPAA------------PWRRFP-
AtZIP9         NGGLMRGAKAFAAGVILATGFVHMLSGGSKALSDPC-LPEF------------PWKMFP-
```

```
OsZIP10         SGGLFVLAKAFAAGVILATGFVHMLHDAEHALSNPC-LPHS------------PWRRFP-
AtIRT3          DGNLFVTAKAFAAGVILATGFVHMLAGGTEALKNPC-LPDF------------PWSKFP-
AtZIP4          EGNLFVAAKAFAAGVILATGFVHMLAGGTEALSNPC-LPDF------------PWSKFP-
AtZIP12         ESNFFMYVKAFAAGVILATGFVHILPDATESLTSSC-LGEEP-----------PWGDFP-
AtZIP3          ETCFFFVTKAFAAGVILATGFMHVLPEAYEMLNSPC-LTSE-------------AWEFP-
AtZIP5          ETTFFFVTKAFAAGVILATGFMHVLPEGYEKLTSPC-LKGE-------------AWEFP-
AtZIP1          ENDIFFMVKAFAAGVILCTGFVHILPDAFERLSSPC-LEDT------------TAGKFP-
OsZIP4          DGDVFFAVKAFAAGVILATGMVHILPAAFDALASPC-GGGRG-----------GGGGFP-
OsZIP3          DGDIFFAVKAFAAGVILATGMVHILPAAFDALTSPC-LKRGG----------GDRNPFP-
si010411m       DGDIFFAVKAFAAGVILATGMVHILPAAFDGLTSPC-LYKVG----------RDRNVFP-
OsZIP8          DGDVFFLVKAFAAGVILATGFIHILPDAFDNLTDDC-LPAGG-----------PWKEFP-
OsZIP5          ETDVFLSVKAFAGGVILATGLVHILPAAFEALSSPC-LV-GG-----------PWKRFP-
OsZIP9          DTSLFFALKAFAAGVILATAFVHILPVSFDKLGSPC-LV-DG-----------PWRKYP-
si024505m       DTDLFIAVKAFAAGVILATAFVHILPDAFEKLGSPC-LV-DG-----------PWQKFP-
                           ..* :*  . :* *  .                             .

AtIAR1          QDLSVGLSVLAGIVVFLLVEKLVRYVEENSSGSNTWGHHHHHHHAG--------------
OsIAR1          EDLSIGLSVLFGIVFFFIVEKIVRYVEDNSQKGAHGMGHHH-HHHK--------------
si013901m       KDLSVGLSILFGIVLFFIVEKIVRYVEDNSQNGAHSMGHGHHHHHK--------------
AtZIP11         ---FAYMLACAGFMLTMLADSVIAHIYSKTQNDLEL------------------------
OsZIP2          ---FAFMLACAGYVLTMLADCAISFVVARGGGRTEPAAAAGAGLEE--------------
Si035517m       ---WAFMLACAGYVVTTLADVVVSHVVSRGRTA-PGSSAGGAELEE--------------
AtZIP2          ---YAFMLAAAGYCLTMLADVAVAFVAAGSNNNHVGASV-GE--SR--------------
OsZIP1          ---FSFMLTCVGFLLTMLSDLVIAAVARRSAAAGVSDNQVSEQQQR--------------
AtZIP6          ---FAGLVTMIGAITALLVDLTASEHMGHGGGGGGDGG-----------MEYMPVG--KA
OsZIP6          ---FAGLFSLVGALLALLVDLSASSHLEAHGHHQHAEEGESPPPPPPTHQPYAPIPTTKK
Si010244m       ---FAGLFTLVGALLALLVDLSASSHLEAHGHGGGGGDGHGHGH---QETTYAP--IPKK
AtZIP7          ---FATFITMISALLVLMIESFAMCAYARRTSKREGEV-------V-----P--------
OsIRT2          ---FAAFVAMLAAVFTLMVDSLMLTFHTRGSKGRAS-------SAV----AH--------
OsIRT1          ---FAAFVAMLAAVSTLMADSLMLTYYNRSKPRPS--SGGDVAAVA----DH--------
Si036196m       ---FTAFVAMLAALFTLMVDSLMLTFYNRKRSGGGNTSGRRAGAAV----AD--------
AtZIP10         ---FAGFVAMMSAVFTLMVDSITTSVFTKSGRKDLRA-------DV----AS--------
AtIRT2          ---FAGFVAMMSGLVTLAIDSITTSLYTGKNSVGPVP-------DE----EY--------
AtIRT1          ---FSGFLAMLSGLITLAIDSMATSLYTSKNAVGIMP-------HG----HG--------
AtZIP8          ---FTGFVAMLSGLVTLAIDSIATSLYTKKAVADDSE-------ER----TT--------
OsZIP7          ---FPGFVAMSAALATLVLDFLATRFYEGKHRAETERVKAAAAAAL----AA-SSASDDD
Si022298m       ---FPGFVAMAAALATLVLDFLATRFYETKHRDEAARVKAAAAATL----AAASSASDED
AtZIP9          ---FPEFFAMVAALLTLLADFMITGYYERKQEKMMNQSVESLGT----------------
OsZIP10         ---FPGFVAMLAALATLVVDFVGTHFYERKHRQEEAAAAAEEAAA--------A--LLED
AtIRT3          ---FPGFFAMIAALITLFVDFMGTQYYERKQEREASESVEPF--G--------R--EQSP
AtZIP4          ---FPGFFAMVAALATLLVDFMGTQYYERKQERNQAATEAAA--G--------S--EE--
AtZIP12         ---MTGLVAMAASILTMLIESFASGYLNRSRLAKEGKTLPVS------------------
AtZIP3          ---FTGFIAMIAAILTLSVDTFATSSFYKSHCKASKRVSDGET-----------------
AtZIP5          ---FTGFIAMVAAILTLSVDSFATSYFHKAHFKTSKRIGDGEE-----------------
AtZIP1          ---FAGFVAMLSAMGTLMIDTFATGYYKRQHFSNNHGSKQVNV-----------------
OsZIP4          ---FAGLVAMAAAMATMMIDSVAAGYYRRSHFKKPRPVDDPAD-----------------
OsZIP3          ---FAGLVSMSAAVSTMVVDSLAAGYYHRSQFRKARPVDNINV-----------------
si010411m       ---FAGLIAMSAAMATMVIDSLAAGYYRRSHFKKARPIDNLEI-----------------
OsZIP8          ---FAGFGAMVGAIGTLVVDTLATGYFTRALSKKDAATAAAVADEE----KQSA------
OsZIP5          ---FAGMVAMVSAIGTLIVDTVATGYFHRTDAKRKAAAVA-DEPAD----DLEA------
OsZIP9          ---FTGLVAMLAAVATLLLDTIATGYFLQRAQDSRGAVAAVAACGG----DASS------
si024505m       ---FTGLVAMLAAIATLVVDTIATGYFQRAHSAKTAAAPV----IG----DVEA------
                      :    .       :

AtIAR1          ------------SKKLKDEGDHNNL-------D---------------QQSSSDAIVNSS
OsIAR1          ---------------RHDRSDKAKL-------NHAEKDHEDKGVNQAEKEPSHDGAIEKT
si013901m       ---------------RHDSSDKAKL-------NHQKSDGDG--------------S----
AtZIP11         ------------------------------------------------------------
OsZIP2          -------------GKLSS-TNGNAS-----------------------------------
Si035517m       -------------GKVSA-TNGTSS-----------------------------------
AtZIP2          -------------E---D-DDV-AV-----------------------------------
OsZIP1          -------------Q---Q-AEG-AV-----------------------------------
AtZIP6          V------GGLEM------------------------------------------------
OsZIP6          SPVFELSGEMSPKKRAHSD----------------DTDRDDVALFGA---KSA---V-RS
Si010244m       APVFELTGEMSPKKRAFLDDDQGDP-APHVFRNGADTDRDDVALFGA---KKGAALV-RS
AtZIP7          -------------------LE-------------------------------------N-
OsIRT2          -------------------H---------------------------------------G
OsIRT1          -------------------GE-------------------------------------SP
Si036196m       -------------------HE-------------------------------------SP
AtZIP10         -------------------VE-------------------------------------TP
AtIRT2          -------------------GI-------------------------------------DQ
AtIRT1          -------------------H----------------------------------------
```

```
AtZIP8       -------------------P----------------------------------------
OsZIP7       ITVVTVTEDD-ND---------NK-----------------APLLQ----PHSH------
Si022298m    ITVVTVAED---D---------RK-----------------APLLQ----THCH------
AtZIP9       -QVSVMSDPGLESGFLRDQEDGGALHIVGMR-AHAEHHRHSLSMGA----EGFEALS-KR
OsZIP10      GGALPV-GDG--EGRDGRGGKRDAMHIVGIH-AHAAAHRHSHAHVH----GACHGGA-VN
AtIRT3       GIVVPMIGEGTNDGKVFGEEDSGGIHIVGIH-AHAAHHRHSHPPGH----DSCEGHS-KI
AtZIP4       IAVVPVVGERVTDNKVFGEEDGGGIHIVGIR-AHAAHHRHSHSNSH----GTCDGH----
AtZIP12      -------------------TGGEEEHAHTGS-AHT--------------------------
AtZIP3       -------------GESS-------------------------------------------
AtZIP5       -------------QDAGGGGGGGDELGLHVH-----------------------------
AtZIP1       -------------------VVDEEEHAGHVH-IHT-------------------------
OsZIP4       ------------AARAAGVEEGGAEHAGHVH-VHT-------------------------
OsZIP3       ------------HKHAG--DE-RAEHAQHIN-AHT-------------------------
si010411m    ------------HEQPG--DEERTGHAQHVH-VHT-------------------------
OsZIP8       -AATQQHNHHHNHHVVGDGGGGGEEHEGQVH-VHT-------------------------
OsZIP5       ------------------------------S-D---------------------------
OsZIP9       --------------------------------SH--------------------------
si024505m    ------------------------------S-DHA-------------------------


AtIAR1       EKVSGGSTDKSLRKRKTSAS--DA-TDKSDSGTE----ITSDGKSDKPEQVETRSSSLVF
OsIAR1       DGVTRADSKSAIRKRGLSSG--SNSADREPVNSES---DPAPNKALSSEDSSVSNSNMVF
si013901m    ----LHESEATIRKRSSSGS--TKATDGEPANSEN---HPAPDKALSSDVSSTSNSNLVF
AtZIP11      --------------QGEDKS--NQ----RSA---------------------TTETSIGD
OsZIP2       DPPAA-------DAAAQDHS--VA----SML---------------------RNASTLGD
Si035517m    EPQPA-------EAHGSDHS--VA----SML---------------------HNANTLGD
AtZIP2       KEEGR-------REIKSGVD--VS----QAL---------------------IRTSGFGD
OsZIP1       -MSRK-------EEEAAAVA--HP----AML---------------------VRTSSFED
AtZIP6       -------------KEGKC-G--AD---L-------------EIQENSEEEIVKMKQRLV
OsZIP6       DEVVV-------APRVGCHG--HH-DVVE-----------VGEEGGGGEEEEARRKQKMV
Si010244m    DEVAV--------VGGGCHG--GGHEVLE-----------VV-GEGAGEEEEARRKQKMV
AtZIP7       -----------------GSNSVDTQNDIQTLENGSSYVEKQE--KVNEDKTSELLRNKVI
OsIRT2       D-------------HGHCHAHALGQADVAALSTTEAADQGSGDVEAGNTTKAQLLRNRVI
OsIRT1       DQ--G-------HRHGHGHGHGHGM--------------AVAKPDDVEATQVQLRRNRVV
Si036196m    AH--G-------HWHGHGHGHGHGHGDIVVAESG-----AVAKPDDDEARKVQLSRNRVV
AtZIP10      DQEIG-------HVQVHGHVHSHT----LPHNL-------HGENDKELGSYLQLLRYRIL
AtIRT2       E-KAI-------HMVGHNHSHGHG----VVL---------------ATKDDGQLLRYQVI
AtIRT1       -----------------GHGPAND----VTLPI-------K----EDDSSNAQLLRYRVI
AtZIP8       -----------------MIIQIDH----LPLTT-------KE---RSSTCSKQLLRYRVI
OsZIP7       ----S-------HSHPHGHGHGHELA--QP------------EGSGGEGEVPAQVRSVVV
Si022298m    ----G-------HSHGHGHNHGHGHE--LV------------QVEGREGDMSDHVRSVVV
AtZIP9       S-------------GVSGHGHGHSHG--H-----------------GDVGLDSGVRHVVV
OsZIP10      D---A-------HAHG----HGHGHE--E-----------------G-----PSARHVVV
AtIRT3       DIGHA-------HAHGHGHGHGHGHV--H-----------------GGLDAVNGARHIVV
AtZIP4       -----------------AHGHSHGHM--H-----------------GNSDVENGARHVVV
AtZIP12      HASQG-------HSHGSLLI--------------------------PQDDDHIDMRKKIV
AtZIP3       ----------------------------------------------VDSEKVQILRTRVI
AtZIP5       --AHG-------HTHGIVGV-------------------------ESGESQVQLHRTRVV
AtZIP1       HASHG-------HTHGST----------------------------------ELIRRRIV
OsZIP4       HATHG-------HAHGHVHSHGHGHG--HS--HGSA---PAAATSPEDASVAETIRHRVV
OsZIP3       HGGHT-------HSHGDIVV----------------------CGSPEEGSVAESIRHKVV
si010411m    HQTQG-------HSHGEVDI----------------------IGSPEEAAIADTIRHRVV
OsZIP8       HATHG-------HAHGSSALVAA----------------------VGEDDKETTLRHRVI
OsZIP5       EHSHG-------HAHGMSVMSV------------------------APAGEEDLVRHRVI
OsZIP9       DHERG-------NAHGVSSAVIASAT--MP--N------DAADDCDDAEDRAKLVRHRVI
si024505m    HGGHG-------HAHGVVSVMAS--------------------TSNADGGGAQLIRHRVI


AtIAR1       GYLNLFSDGVHNFTDGMALGSAFLIYGSVGGWSRTMFLLAHELPQEIGDFGILVRS--GF
OsIAR1       GYLNLFSDGVHNFTDGMALGSAFLLHGSVGGWSRTLFLLAHELPQEVGDFGILVRS--GF
si013901m    GYLNLFSDGVHNFTDGMALGSAFLLQGSVGGWSRTLFLLAHELPQEVGDFGILVRS--GF
AtZIP11      SILLIVALCFHSVFEGIAIGISE---TKSDAWRALWTITLHKIFAAIAMGIALLRMIPDR
OsZIP2       SVLLIAALCFHSVFEGIAIGVAE---TKADAWKALWTISLHKIFAAIAMGIALLRMLPDR
Si035517m    SILLIAALCFHSVFEGIAIGVAE---TKADAWKALWTISLHKIFAAIAMGIALLRMLPNR
AtZIP2       TALLIFALCFHSIFEGIAIGLSD---TKSDAWRNLWTISLHKVFAAVAMGIALLKLIPKR
OsZIP1       AVLLIVALCFHSVFEGIAIGVSA---SKSEAWRNLWTIGLHKIFAAVAMGIALLRMIPKR
AtZIP6       SQVLEIGIIFHSVIIGVTMGMSQ---NKCTIRPLIAALSFHQIFEGLGLGGCIAQA--GF
OsZIP6       SKVLEIGIVFHSVIIGVTMGMSQ---DVCAIRPLVVALSFHQVFEGMGLGGCIAQA--GF
Si010244m    SKVLEIGIVFHSVIIGVTMGMSQ---DVCAIRPLVVALSFHQVFEGMGLGGCIAQA--GF
AtZIP7       AQILELGIVVHSVVIGLAMGASD---NKCTVQSLIAALCFHQLFEGMGLGGSILQA--QF
OsIRT2       VQVLEMGIVVHSVVIGLGMGASQ---NVCTIRPLVAALCFHQMFEGMGLGGCILQA--GY
OsIRT1       VQVLEIGIVVHSVVIGLGMGASQ---NVCTIRPLVAAMCFHQMFEGMGLGGCILQA--EY
```

```
Si036196m      VQVLEMGIIVHSVVIGLGMGASQ---SVCTIRPLVAAMCFHQLFEGMGLGGCILQA--EY
AtZIP10        AIVLELGIVVQSIVIGLSVGDTN---NTCTIKGLVAALCFHQMFEGMGLGGCILQA--EY
AtIRT2         AMVLEVGILFHSVVIGLSLGATN---DSCTIKGLIIALCFHHLFEGIGLGGCILQA--DF
AtIRT1         AMVLELGIIVHSVVIGLSLGATS---DTCTIKGLIAALCFHQMFEGMGLGGCILQA--EY
AtZIP8         ATVLELGIIVHSVVIGLSLGATN---DTCTIKGLIAALCFHQMFEGMGLGGCILQA--EY
OsZIP7         SQILEMGIVSHSVIIGLSLGVSR---SPCTIRPLVAALSFHQFFEGFALGGCIAQA--QF
Si022298m      SQILEMGIVSHSVIIGLSLGVSR---SPCTIRPLVAALSFHQFFEGFALGGCIAQA--QF
AtZIP9         SQILEMGIVSHSIIIGISLGVSH---SPCTIRPLLLALSFHQFFEGFALGGCVAEA--RL
OsZIP10        SQILELGIVSHSVIIGLSLGVSQ---SPCTIKPLVAALSFHQFFEGFALGGCISEA--QL
AtIRT3         SQVLELGIVSHSIIIGLSLGVSQ---SPCTIRPLIAALSFHQFFEGFALGGCISQA--QF
AtZIP4         SQILELGIVSHSIIIGLSLGVSQ---SPCTIRPLIAALSFHQFFEGFALGGCISQA--QF
AtZIP12        TQILELGIVVHSVIIGISLGASP---SVSTIKPLIAAITFHQLFEGFGLGGCISEA--KF
AtZIP3         AQVLELGIIVHSVVIGISLGASQ---SPDAAKALFIALMFHQCFEGLGLGGCIAQG--KF
AtZIP5         AQVLEVGIIVHSVVIGISLGASQ---SPDTAKALFAALMFHQCFEGLGLGGCIAQG--NF
AtZIP1         SQVLEIGIVVHSVIIGISLGASQ---SIDTIKPLMAALSFHQFFEGLGLGGCISLA--DM
OsZIP4         SQVLELGILVHSVIIGVSLGASL---RPSSIRPLVGALSFHQFFEGIGLGGCIVQA--NF
OsZIP3         SQVLELGILVHSVIIGVSLGASV---RPSTIRPLVGALSFHQFFEGVGLGGCIVQA--NF
si010411m      SQVLELGILVHSVIIGVSLGTSV---RSSTIRPLVGALSFHQLFEGIGLGGCIVQA--NF
OsZIP8         SQVLELGIVVHSVIIGISLGASQ---NPETIKPLVVALSFHQMFEGMGLGGCIVQA--KF
OsZIP5         SQVLELGVVVHSLIIGMSLGASD---FPSTVRPLVPALTFHQFFEGIGLGGCIVQA--KF
OsZIP9         SQVFELGIIVHSIIIGISLGASE---SPSTIRPLVAALTFHQFFEGIGLGGCIVQA--RF
si024505m      AQVLELGIIVHSVIIGMSVGASE---SPSTIRPLVAALTFHQFFEGIGLGGCIVQA--KF
                 :   .   :..  *: :* :               :  *.    ..    :

AtIAR1         TV----TKALFFNFLSALVALAGTALVLVWGNEPG------QSSLIEGFTAGGFIYIAVA
OsIAR1         TV----TKALFFNFLSALVALAGTALALSLGKDPG------HSSLIEGFTAGGFIYIAVA
si013901m      SV----SKALFFNFLSALVALAGTALALSLGKDPG------HSSLIEGFTAGGFIYIAVA
AtZIP11        PLFSSITYSFAFAISSPIGVAIGIVIDATT----QGSIADWIFALSMSLACGVFVYVSVN
OsZIP2         PFLSCFGYAFAFAVSSPVGVGIGIVIDATT----QGRVADWIFAVSMGLATGIFIYVSIN
Si035517m      PFLSCFAYAFAFAISSPIGVAIGIVIDATT----QGRVADWIFAISMGLATGIFVYVSIN
AtZIP2         PFFLTVVYSFAFGISSPIGVGIGIGINATS----QGAGGDWTYAISMGLACGVFVYVAVN
OsZIP1         PFLMTVVYSLAFAVSSPVGVGIGIAIDATS----QGRAADWTYAISMGLATGVFIYVAIN
AtZIP6         KAGTVVYMCLMFAVTTPLGIVLGMVIFAATGYDDQNPNALIMEGLLGSFSSGILIYMALV
OsZIP6         GIATVGYMCVMFSVTTPLGILLGMAIFHMTGYDDSSPNALIIEGLLGSLSSGILVYMALV
Si010244m      GMATVGYMCIMFSVTTPLGILLGMLIFHMTGYDDSNPNALIMEGILGSLSAGVLIYMALV
AtZIP7         KSKTNWTMVFFFSVTTPFGIVLGMAIQKI--YDETSPTALIVVGVLNACSAGLLIYMALV
OsIRT2         GGRTRSALVFFFSTTTPFGIALGLALTRV--YSDSSPTALVVVGLLNAASAGLLHYMALV
OsIRT1         GRRMRSVLVFFFSTTTPFGIALGLALTRV--YRDNSPTALIVVGLLNAASAGLLHYMALV
Si036196m      GLKMKSGLVFFFSTTTPFGIALGLALTRV--YRENSPTALIVVGILNAASAGLLHYMALV
AtZIP10        GWVKKAVMAFFFAVTTPFGVVLGMALSKT--YKENSPESLITVGLLNASSAGLLIYMALV
AtIRT2         TNVKKFLMAFFFTGTTPCGIFLGIALSSI--YRDNSPTALITIGLLNACSAGMLIYMALV
AtIRT1         TNMKKFVMAFFFAVTTPFGIALGIALSTV--YQDNSPKALITVGLLNACSAGLLIYMALV
AtZIP8         TNVKKFVMAFFFAVTTPSGIALGIALSSV--YKDNSPTALITVGLLNACSAGLLIYMALV
OsZIP7         KTLSAAIMACFFAITTPAGIAAGAGVASF--YNANSPRALVVEGILDSVSAGILIYMSLV
Si022298m      KNLSAVLMASFFAITTPAGIAAGAGLATF--YNPNSPRALVVEGILDSVSAGILIYMSLV
AtZIP9         TPRGSAMMAFFFAITTPIGVAVGTAIASS--YNSYSVAALVAEGVLDSLSAGILVYMALV
OsZIP10        KNFSAFLMAFFFAITTPAGITVGAAVASF--YNPNSPRALVVEGILDSMSAGILIYMALV
AtIRT3         RNKSATIMACFFALTTPIGIGIGTAVASS--FNSHSVGALVTEGILDSLSAGILVYMALV
AtZIP4         RNKSATIMACFFALTTPLGIGIGTAVASS--FNSHSPGALVTEGILDSLSAGILVYMALV
AtZIP12        RVKKIWVMLMFFALTAPIGIGIGIGVAEI--YNENSPMALKVSGFLNATASGILIYMALV
AtZIP3         KCLSVTIMSTFFAITTPIGIVVGMGIANS--YDESSPTALIVQGVLNAASAGILIYMSLV
AtZIP5         NCMSITIMSIFFSVTTPVGIAVGMAISSS--YDDSSPTALIVQGVLNAASAGILIYMSLV
AtZIP1         KSKSTVLMATFFSVTAPLGIGIGLGMSSGLGYRKESKEAIMVEGMLNAASAGILIYMSLV
OsZIP4         KAKATVIMATFFSLTAPVGIALGIAISSS--YSKHSSTALVVEGVFNSAAAGILIYMSLV
OsZIP3         KVRATVIMAIFFSLTAPVGIVLGIAISSS--YNVHSSTAFVVEGVFNSASAGILIYMSLV
si010411m      KLRATVMMAIFFSLTAPIGIALGIGISSS--YNGHSATAFVVEGVFNSASAGILIYMSLV
OsZIP8         KVRSIVTMVLFFCLTTPVGIAVGVGISSV--YNESSPTALVVEGILNSVAAGILIYMALV
OsZIP5         RVRSVVTMALFFSLTTPAGIVVGIGISSV--YDANSPTALVVQGLLEAAAAGILVYMALV
OsZIP9         HLKSAVTMAIFFSLTTPVGIMIGIGISSA--YNENSPTALIVEGILDAAAAGILNYMALV
si024505m      RLKSMLTMALFFSLTTPVGVVIGIGISST--YNENSPRALIVEGVLNAAAAGILNYMALV
                          *   :      *  :                 ..  . : * : *:::

AtIAR1         GVLAEMNNSGKSTL----KNSACHLISLILGMSVALCISLIE----
OsIAR1         GVLPEMNDQ-KTTV----KSSMIQLVSLTMGMLVALGISLVE----
si013901m      GVLPQMNDQ-KTTL----KSSVVQLISLAMGMLVALGISLVE----
AtZIP11        HLLAKGYRPNKKV------HVDEPRYKFLAVLFGVVVIAIVMIWDT
OsZIP2         HLLSKGYTPLRPV------AADTPAGRLLAVVLGVAVIAVVMIWDT
Si035517m      HLLSKGYKPRRPV------AVDTPVGRWLAVVLGVAVIAVVMIWDT
AtZIP2         HLISKGYKPREEC------YFDKPIYKFIAVFLGVALLSVVMIWD-
OsZIP1         HLIAKGYRPHHPT------AADKPLFKFLAVLLGVAVMAVVMIWD-
AtZIP6         DLIALDFFHNKMLTTCGESGSRLKKLCFVALVLGSASMSLLALWA-
OsZIP6         DLISLDFFHNKMMSS----SNKLKKVSYVALVLGSASMSILALWA-
```

```
Si010244m      DLISLDFFHNKMMSA----SNKLKKACYIALVLGSASMSILALWA-
AtZIP7         NLLAHEFFGPKIQG-----NIKLHVLGYVATFTGAAGMSLMAKWA-
OsIRT2         ELLAADFMGPKLQG-----NVRLQLAASLAILLGAGGMSVMAKWA-
OsIRT1         ELLAADFMGPKLQG-----NVRLQLAAFLAVLLGAGGMSVMAKWA-
Si036196m      ELLAADFMGPKLQG-----SVRLQLVSFAAVLLGAGGMSVMAKWA-
AtZIP10        DLLAADFMGQKMQR-----SIKLQLKSYAAVLLGAGGMSVMAKWA-
AtIRT2         DLLATEFMGSMLQG-----SIKLQIKCFTAALLGCAVMSVVAVWA-
AtIRT1         DLLAAEFMGPKLQG-----SIKMQFKCLIAALLGCGGMSIIAKWA-
AtZIP8         DLLAAEFMGSMLQR-----SVKLQLNCFGAALLGCGGMSVLAKWA-
OsZIP7         DLIAADFLGGKMTG-----STRQQVMAYIALFLGALSMSSLAIWA-
Si022298m      DLIAADFLGEKMTG-----SLRQQLVAYIALFLGALSMSSLAIWA-
AtZIP9         DLIAADFLSKKMSV-----DFRVQVVSYCFLFLGAGMMSALAIWA-
OsZIP10        DLIAADFLSRKMSC-----NPRLQVGSYIALFLGAMAMAALALWA-
AtIRT3         DLIAADFLSTKMRC-----NFRLQIVSYVMLFLGAGLMSSLAIWA-
AtZIP4         DLIAADFLSKRMSC-----NLRLQVVSYVMLFLGAGLMSALAIWA-
AtZIP12        DLVAPLFMNQKTQS-----SMKIQVACSVSLVVGAGLMSLLAIWA-
AtZIP3         DLLAADFTHPKMQS-----NTGLQIMAHIALLLGAGLMSLLAKWA-
AtZIP5         DFLAADFMHPKMQS-----NTRLQIMAHISLLVGAGVMSLLAKWA-
AtZIP1         DLLATDFMNPRLQS-----NLWLHLAAYLSLVLGAGSMSLLAIWA-
OsZIP4         DLLAADFNNPKLQT-----NTKLQLAVYLALFLGAGMMSLLAIWA-
OsZIP3         DLLATDFNNPKLQI-----NTKLQLMAYLALFLGAGLMSMLAIWA-
si010411m      DLLATDFNNPKLQT-----NTKLQLMTYLALFLGAGMMSMLAIWA-
OsZIP8         DLLAEDFMNPRVQS-----KGKLQLGINLAMLAGAGLMSMLAKWA-
OsZIP5         DILAEDFMKTKVQR-----RGRLQLAMNVALLLGAGLMSMIAIWA-
OsZIP9         DLLAEDFMNPRVRK-----SGRLQLIISILLLVGIALMSLLGIWA-
si024505m      DLLAEDFMNPRVQN-----NGRLQVIVSVSLLVGAALMSMLAIWA-
                .:                            .     :: :
```

PLEASE NOTE: Showing colors on large alignments is slow.
