# Supplementary material for: Genome Wide Identification of Orthologous ZIP Genes Associated with Zinc and Iron Translocation in Setaria italica
Source: Front Plant Sci. 2017 May 15;8:775. doi: 10.3389/fpls.2017.00775 (PMC5430159; doi:10.3389/fpls.2017.00775)
Supplement: Supplementary file 6 [file Table_1.DOCX]

**Table S1: List of primers used for the expression analysis of SiZIP family gene**

| **No** | **Gene id** | **5'<--Sequence-->3'** | **SEQUENCE** | **Length** | **Amplicon**  **Length** |
| --- | --- | --- | --- | --- | --- |
| 1 | Si036196m.g | F | CAACCTCTTCGTCATCGTCAAG | 22 | 216 |
|  |  | R | CTCTTCCGGTTGTAGAAGGTGAG | 23 |  |
| 2 | Si022298m.g | F | GATGGGGATTGTGTCTCACTCT | 22 | 214 |
|  |  | R | CTATCCCTGCCGGTGTTGTAAT | 22 |  |
| 3 | Si035517m.g | F | CACCTCCTCATCTGCCTCTC | 20 | 218 |
|  |  | R | GTTCCAGCGCATGAAGTAGG | 20 |  |
| 4 | Si010244m.g | F | CATGAGGTGTTGGAGGTGGT | 20 | 195 |
|  |  | R | GAACACCTGGTGGAAGGAGAG | 21 |  |
| 5 | Si013901m.g | F | ACTCTAACTTGGTGTTCGGCTACC | 23 | 200 |
|  |  | R | GGCCTTAGATACTGAGAACCCTGA | 24 |  |
| 6 | Si024505m.g | F | TCTGTAGGTGCATCTGAGAGTCC | 23 | 145 |
|  |  | R | AGAAGAAGAGGGCCATTGTCAG | 22 |  |
| 7 | Si010411m.g | F | GGAGTGTCCTTAGGTACATCTGTG | 24 | 175 |
|  |  | R | CTATGGGTGCAGTTAGGGAGAA | 22 |  |
