# Supplementary material for: Genome Wide Identification of Orthologous ZIP Genes Associated with Zinc and Iron Translocation in Setaria italica
Source: Front Plant Sci. 2017 May 15;8:775. doi: 10.3389/fpls.2017.00775 (PMC5430159; doi:10.3389/fpls.2017.00775)
Supplement: Supplementary file 7 [file Table_2.DOCX]

**Table S2: ZIP family genes and cleavage site of signal peptide details across Rice, Arabidopsis and Foxtail millet.**

| **Gene** | **Source Accession no.** | **Chromosome coordinates** | **Protein length (aa)** | **p I** | **Mw (Daltons)** | **Sub cellular localization** | **Signal peptide cleavage site at amino acid- Position** | **No of TM domains** |
| --- | --- | --- | --- | --- | --- | --- | --- | --- |
| AtZIP1 | AT3G12750 | 3: 4,051,651-4,053,201 | 355 | 6.70 | 37811.8 | SP | 28 and 29 | 8 |
| AtZIP2 | AT5G59520 | 5: 23,991,253-23,992,798 | 353 | 6.39 | 38312.1 | SP | 27 and 28 | 9 |
| AtZIP3 | AT2G32270 | 2: 13,704,221-13,706,860 | 339 | 7.26 | 36043.0 | SP | 25 and 26 | 8 |
| AtZIP4 | AT1G10970 | 1: 3,665,087-3,667,139 | 408 | 6.59 | 43170.3 | --- | --- | 6 |
| AtZIP5 | AT1G05300 | 1: 1,545,106-1,547,849 | 360 | 6.26 | 38167.4 | SP | 26 and 27 | 8 |
| AtZIP6 | AT2G30080 | 2: 12,838,730-12,840,112 | 341 | 5.71 | 36021.5 | --- | --- | 8 |
| AtZIP7 | AT2G04032 | 2: 1,289,832-1,291,383 | 365 | 5.90 | 39,378.0 | SP | 26 and 27 | 8 |
| AtZIP8 | AT5G45105 | 5: 18,223,453-18,224,946 | 347 | 6.64 | 37197.2 | SP | 27 and 28 | 8 |
| AtZIP9 | AT4G33020 | 4: 15,932,603-15,934,267 | 344 | 6.18 | 36151.1 | --- | --- | 6 |
| AtZIP10 | AT1G31260 | 1: 11,175,559-11,177,362 | 364 | 8.40 | 39489.4 | SP | 28 and 29 | 8 |
| AtZIP11 | AT1G55910 | 1: 20,906,134-20,907,413 | 326 | 5.67 | 35461.4 | SP | 20 and 21 | 8 |
| AtZIP12 | AT5G62160 | 5: 24,960,107-24,961,263 | 355 | 6.79 | 37571.2 | SP | 25 and 26 | 8 |
| OsZIP1 | OS01G0972200 | 1: 42,905,570-42,907,462 | 352 | 8.90 | 37446.9 | SP | 29 and 30 | 8 |
| OsZIP2 | OS03G0411800 | 3: 17,008,027-17,011,263 | 358 | 5.70 | 36645.4 | MT | 27 and 28 | 8 |
| OsZIP3 | OS04G0613000 | 4: 31,078,201-31,080,558 | 364 | 8.82 | 38134.4 | SP | 23 and 24 | 7 |
| OsZIP4 | OS08G0207500 | 8: 6,267,823-6,270,904 | 396 | 8.32 | 39965.4 | SP | 29 and 30 | 7 |
| OsZIP5 | OS05G0472700 | 5: 23,216,442-23,218,934 | 353 | 6.35 | 36754.2 | SP | 27 and 28 | 8 |
| OsZIP6 | OS05G0164800 | 5: 3,807,974-3,810,780 | 395 | 6.34 | 41326.1 | --- | --- | 8 |
| OsZIP7 | OS05G0198400 | 5: 6,090,801-6,094,068 | 384 | 6.56 | 39727.8 | MT | --- | 6 |
| OsZIP8 | OS07G0232800 | 7: 7,394,486-7,397,577 | 390 | 6.30 | 40256.5 | SP | 25 and 26 | 8 |
| OsZIP9 | OS05G0472400 | 5: 23,201,237-23,212,773 | 362 | 5.97 | 37896.3 | SP | 21 and 22 | 8 |
| OsZIP10 | OS06G0566300 | 6: 21,824,893-21,826,815 | 404 | 6.53 | 41525.6 | SP | --- | 6 |
| AtIRT1 | AT4G19690 | 4: 10,707,426-10,709,015 | 347 | 6.69 | 36726.7 | SP | 28 and 29 | 8 |
| AtIRT2 | AT4G19680 | 4: 10,703,362-10,704,816 | 350 | 6.39 | 37407.2 | SP | 21 and 22 | 8 |
| AtIRT3 | AT1G60960 | 1: 22,445,310-22,447,214 | 445 | 6.19 | 45089.0 | SP | --- | 6 |
| OsIRT1 | OS03G0667500 | 3: 26,286,254-26,292,018 | 374 | 8.89 | 39057.0 | SP | 26 and 27 | 8 |
| OsIRT2 | OS03G0667300 | 3: 26,276,422-26,277,614 | 370 | 8.67 | 38269.8 | SP | 25 and 26 | 8 |
| AtIAR1 | AT1G68100 | 1: 25,521,204-25,524,175 | 469 | 5.84 | 50582.8 | SP | 28 and 29 | 6 |
| OsIAR1 | OS08G0467400 | 8: 22,987,096-22,994,967 | 498 | 6.20 | 53578.7 | SP | 20 and 21 | 6 |
| SiZIP1 | Si022298m.g | 3: 7595453-7598254 | 389 | 6.99 | 40760.09 | MT | --- | 6 |
| SiZIP2 | Si024505m.g | 3: 16273185-16276946 | 354 | 6.04 | 36810.17 | SP | 25 and 26 | 8 |
| SiZIP3 | Si013901m.g | 6: 30177843-30185502 | 390 | 6.03 | 41682.85 | --- | --- | 6 |
| SiZIP4 | Si010411m.g | 7: 29716578-29718761 | 367 | 7.28 | 38857.31 | SP | 23 and 24 | 7 |
| SiZIP5 | Si010244m.g | 7: 32421561-32424492 | 406 | 6.26 | 42120.95 | --- | --- | 8 |
| SiZIP6 | Si036196m.g | 9: 9488234-9492357 | 377 | 8.86 | 39599.47 | SP | 23 and 24 | 8 |
| SiZIP7 | Si035517m.g | 9: 42693597-42697136 | 471 | 9.77 | 50123.03 | --- | --- | 8 |
