# Supplementary material for: Genome Wide Identification of Orthologous ZIP Genes Associated with Zinc and Iron Translocation in Setaria italica
Source: Front Plant Sci. 2017 May 15;8:775. doi: 10.3389/fpls.2017.00775 (PMC5430159; doi:10.3389/fpls.2017.00775)
Supplement: Supplementary file 8 [file Table_3.DOCX]

**Table S3: SSR mining of the SiZIP family gene**

| **Gene** | **Repeat motif** | **Position** |
| --- | --- | --- |
| SiZIP1 | -- | -- |
| SiZIP2 | (TC)_6_ | Intronic |
| SiZIP3 | (CCA)_5_ | CDS |
| SiZIP4 | -- | -- |
| SiZIP5 | (TC)_5_ | Intronic |
| SiZIP6 | -- | -- |
| SiZIP7 | (GC)_5_ | Cds |
